# Multi-Omics of *Corynebacterium Pseudotuberculosis* 12CS0282 and an In Silico Reverse Vaccinology Approach Reveal Novel Vaccine and Drug Targets

**DOI:** 10.3390/proteomes10040039

**Published:** 2022-11-23

**Authors:** Jens Möller, Mona Bodenschatz, Vartul Sangal, Jörg Hofmann, Andreas Burkovski

**Affiliations:** 1 Microbiology Division, Department of Biology, Faculty of Sciences, Friedrich-Alexander-Universität Erlangen-Nürnberg, Staudtstr. 5, 91058 Erlangen, Germany; 2 Faculty of Health and Life Sciences, Northumbria University, Newcastle upon Tyne NE1 8ST, UK; 3 Biochemistry Division, Department of Biology, Faculty of Sciences, Friedrich-Alexander-Universität Erlangen-Nürnberg, Staudtstr. 5, 91058 Erlangen, Germany

**Keywords:** caseous lymphadenitis, CLA, corynebacteria, lymphangitis, microbial proteomics, phospholipase D, zoonosis

## Abstract

*Corynebacterium pseudotuberculosis* is an important animal pathogen, which is also able to infect humans. An optimal treatment of infections with this pathogen is not available today and consequently, more research is necessary to understand the infection process. Here, we present a combined -omics and bioinformatics approach to characterize *C. pseudotuberculosis* 12CS0282. The genome sequence of strain 12CS0282 was determined, analyzed in comparison with the available 130 *C. pseudotuberculosis* sequences and used as a basis for proteome analyses. In a reverse vaccinology approach, putative vaccine and drug targets for 12CS0208 were identified. Mass spectrometry analyses revealed the presence of multiple virulence factors even without host contact. In macrophage interaction studies, *C. pseudotuberculosis* 12CS0282 was highly resistant against human phagocytes and even multiplied within human THP-1 cells. Taken together, the data indicate a high pathogenic potential of the strain.

## 1. Introduction

*Corynebacterium pseudotuberculosis* is a zoonotic pathogen closely related to *Corynebacterium ulcerans* and *Corynebacterium diphtheriae*. Based on the fact that these three species can be lsogenized by a corynebacteriophage [1], and as a result may carry the *tox* gene coding for diphtheria toxin (DT), *C. pseudotuberculosis*, *C. ulcerans* and *C. diphtheriae* are forming the group of toxigenic corynebacteria [2]. The species is divided into two biovars based on biochemical properties (e.g., nitrate metabolism), infected host animals and evoked diseases [3]. Biovar *ovis* is the causative agent of caseous lymphadenitis in small ruminants such as sheep and goats and mastitis in dairy cattle [4,5,6], while biovar *equi* causes abscesses as well as ulcerative lymphangitis in equines and oedematous skin disease in buffalos [7].

Due to the importance of host animals for milk, meat, leather and wool production, and the worldwide distribution of the pathogen, significant economic losses are resulting from *C. pseudotuberculosis* infections [3]. Furthermore, as a zoonotic pathogen, humans can also be infected with *C. pseudotuberculosis* causing lymphadenitis [8,9]. Consequently, it is not astonishing that a number of *C. pseudotuberculosis* sequencing and proteome projects have been carried out, beginning in 2010 (e.g., [9,10,11,12]), and often with the aim of identifying candidates for vaccine production (e.g., [13,14,15]). Nevertheless, as concluded in a recent analysis of transcriptional regulatory networks in *C. pseudotuberculosis* [16], more experimental studies are needed for a better understanding of this important animal pathogen.

Here, we present a combined genome and proteome analysis of *C. pseudotuberculosis* strain 12CS0208 isolated from a goat in Thuringia, Germany.

## 2. Materials and Methods

### 2.1. Bacteria and Growth Conditions

*C. pseudotuberculosis* strains 12CS0282 and FRC41, *C. ulcerans* 809, *Corynebacterium glutamicum* ATCC13032 and *Corynebacterium silvaticum* W25 were cultivated at 37 °C on Columbia Blood agar plates (CBA, Oxoid, Wesel, Germany) or in Brain Heart Infusion (BHI, Oxoid, Wesel, Germany) under constant shaking at 125 rpm in baffled flasks.

### 2.2. Genome Sequencing and Analysis

DNA for whole genome sequencing was prepared after cultivation in BHI using QIAGEN Genomic-tips 20/G and a QIAGEN Genomic DNA buffer set kit (Qiagen, Hilden, Germany). DNA was sequenced on an Illumina MiSeq instrument (Illumina, San Diego, CA, USA).

The paired-end sequencing reads were assembled using SPAdes 3.13.1 [17] and scaffolded using MeDuSa [18] with the genome sequence of *C. pseudotuberculosis* strain 29,156 used as the reference. The genome sequences of strains *C. pseudotuberculosis* 12CS0282 has been submitted to GenBank (Accession no.: NZ_CP074370.1).

16S rRNA gene sequence from the genome was extracted using RNAMMER/1.2 [19] and were aligned to 16S rRNA reference sequences for the genus *Corynebacterium* obtained from the GenBank using MUSCLE [20]. A maximum-likelihood tree was constructed from the alignment after removing the sites with the missing data using IQ-Tree following the GTR + F + I + G4 substitution model with 100,000 SH-aLRT and 100,000 ultrafast bootstrap iterations [21]. The tree was visualized using iTol server [22].

Digital DNA-DNA hybridization (dDDH) values were calculated between the genome sequence of *C. pseudotuberculosis* 12CS0282 and *Corynebacterium belfantii* DSM 105776 ^T^ (OANN01), *C. diphtheriae* DSM 44123 ^T^ (LJXR01), *C. pseudotuberculosis* ATCC19410 ^T^ (CP021251.1), *C. pseudotuberculosis* DSM 20689 ^T^ (RBXH01), *C. silvaticum* KL0182 ^T^ (SDQO02), *C. silvaticum* W25 (VFEM01), *C. ulcerans* FRC11 (CP009622.1) and *C. ulcerans* NCTC 7910 ^T^ (LT906443.1) using the GGDC server [23,24].

To compare the genomic variation with proteomic analyses, we obtained sequences of strains *C. pseudotuberculosis* 258 (GCA_000263755.3), *C. silvaticum* W25 (GCA_006370535.1), *C. diphtheriae* C7 (beta; GCA_000255175.1), *C. ulcerans* 809 (GCA_000215645.1), *C. pseudotuberculosis* 1/06A (GCA_000233735.1), *C. pseudotuberculosis* E16 (GCA_008461845.1), *C. pseudotuberculosis* I19 (GCA_000152065.3) and *C. pseudotuberculosis* FRC41 (GCA_000143705.2) from the Genbank. All genomes were annotated using Prokka [25] and were compared using Roary with a sequence identity cut-off of 70% [26,27]. Finally, all available 130 *C. pseudotuberculosis* genomes were also obtained from the GenBank and were compared as mentioned above.

The virulence genes previously reported in *C. pseudotuberculosis* strains [9] were searched across all *C. pseudotuberculosis* genomes using protein BLAST searches [28].

### 2.3. Sample Preparation of Proteomic Analyses

Preparation of whole cell proteins was carried out as described previously [29]. For this purpose, the cells were resuspended in PBS buffer with protease inhibitor (Complete, Roche, Basel, Switzerland) and lysed with a homogenizer (MP Biomedical, Fisher Scientific, Schwerte, Germany) using glass beads (5.5 m s^−1^, 30 s, 5 cycles, 4 °C). Cell debris was removed by centrifugation at 13,000× *g* for 30 min at 4 °C and the supernatant was retained. Protein buffer was added to a final concentration of 10 mM DTT, 2% sodium deoxycholate and 50 mM Tris (pH 8.0). For in solution tryptic digest, 40 µg of protein was transferred into a new low-binding tube and incubated at 55 °C for 30 min for protein denaturation followed by an alkylation of sulfhydryl groups (40 mM CAA final concentration). Precipitation of proteins was carried out overnight at 4 °C with 80% acetone, washed three times with 80% ice-cold acetone, dried under N_2_ atmosphere to avoid oxidation [30] and resuspended in 100 mM TEAB buffer. Proteins were digested overnight at 37 °C under constant shaking with 2 µg of trypsin. Desalting occurred with C18 stage tips with 25 µg of all peptide samples (three biological replicates of secreted proteins, whole proteome and surface proteins). Peptides were vacuum-dried and resuspended in 0.1% trifluoroacetic acid (TFA) for LC-MS/MS analysis [31].

For preparation of extracellular proteins, the bacteria were removed by centrifugation (10 min, 4 °C, 4000× *g*) and the supernatant was subsequently filtered using a 0.2 µm pore size filter (Minisart, Sartorius, Göttingen, Germany). The proteins were precipitated using 10% (*w*/*v*) trichloroacetic acid (TCA) and resuspended in protein buffer (10 mM DTT, 2% sodium deoxycholate, 50 mM Tris, pH 8.0). Pierce^TM^ 660 nm Protein Assay (Thermo Fisher Scientific, Bremen, Germany) was used to determine the protein concentration. 25 µg of the proteins were used for a tryptic digest on 10 kDa vivacon 500 membrane filters as previously described following a modified Filter Aided Sample preparation (FASP) protocol [30,32,33]. Cell surface proteins were isolated by tryptic shaving [31,34]. For this purpose, cells were harvested and treated with 1.5 µg sequencing grade trypsin (Promega, Madison, WI, USA) for 1.5 h at 37 °C.

### 2.4. Mass Spectrometry

Mass spectrometric analyses were carried as described before [29,30,31,33]. The separation of 10 µg of peptides were carried out by a nanoflow Ultimate 3000 HPLC (Dionex, Sunnyvale, CA, USA) using an EASY-Spray column (Thermo Fisher Scientific; C18 with 2 µm particle size, 50 cm × 75 µm) with a (flow rate of 200 nL min^−1^ and increasing acetonitrile concentrations over 120 min. The total method duration including equilibration and column wash was set to 160 min. Triplicates of all samples were analyzed using an Orbitrap Fusion mass spectrometer (Thermo Fisher Scientific, Bremen, Germany) with following settings: spray voltage 2000 V, transfer tube temperature 275 °C, scan range for the MS 1 detection in the Orbitrap 300–2000 (*m*/*z*), 50 ms maximum injection time, automatic gain control (AGC) target of 4 × 10^6^ and Orbitrap resolution of 120.000 [29]. For collision-induced dissociation with a collision energy of 35%, the 10 most intense ions were selected and for ion trap detection a maximum injection time of 250 ms and an AGC target of 1 × 10^5^ were set. Resulting raw data files were analyzed using the Proteome Discoverer 1.4 program package (Thermo Fisher Scientific, Bremen, Germany) and the theoretical proteome of *C. pseudotuberculosis* 12CS0282. As described by Schäfer and co-workers [35], the theoretical masses of peptides were generated with a maximum of two missed cleavages. Carbamidomethyl modification on cysteine was set as fixed modification, oxidation of methionine as dynamic modification. To compare the measured spectra of product ions, the mass tolerance for survey scans was set to 10 ppm and 0.6 Da for fragment mass measurements. False discovery rate (FDR) was set on 1% for protein identification.

### 2.5. Characterization and Visualization of Proteome Data

Localization prediction and detection of signal peptides was carried out using the psortb server [36] and LipoP [37]. Proteins including transmembrane helices were detected with the TMH MM Server v.2.0 tool [38,39]. For pathway analysis, amino acid sequences from the theoretical proteome were used for a BlastKOALA search [40]. Missing information was completed by a proteome comparison, carried out using the PATRIC 3.6.5 database [41] and extracted from existing data of homologous proteins from other *C. pseudotuberculosis* strains. Visualization of the generated data was carried out using the proteomaps program [42,43,44].

### 2.6. Reverse Vaccinology

**Figure 1:**
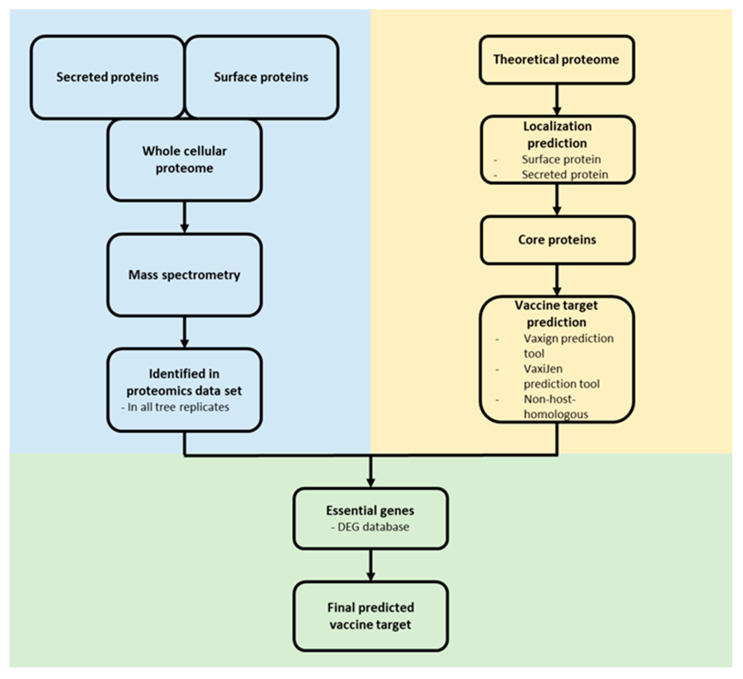
Reverse vaccinology approach. Proteome data (blue) and in silico protein characterization (yellow) were combined to predict potential vaccine and drug targets (green).

For the identification of novel vaccine targets, an in silico reverse vaccinology approach was applied [45,46]. Surface associated and secreted proteins from the core proteome of *C. pseudotuberculosis* were extracted and analyzed for a putative essential function using the DEG 10 database [47]. Only proteins with the following parameters were considered as essential: bit score > 100, E-value with a cut-off of 1 × 10^−4^ and percentage of identity > 35% [48]. The remaining proteins were analyzed as probable vaccine targets and non-host-homologous proteins (human, pig and mouse) were excluded using the Vaxign2 prediction tool [49]. The antigen classification of the proteins was analyzed by VaxiJen [50]. For antigenicity analysis, the default parameter value was set to 0.4. The ProtParam tool [51] estimated the molecular weight (MW) and stability of the proteins (Figure 1).

To visualize protein-protein interaction networks of selected vaccine candidates from the reverse vaccinology approach the Search Tool for the Retrieval of Interacting Genes/Proteins–STRING was used with strain C231 as reference proteome [52].

### 2.7. Interaction of C. Pseudotuberculosis Strain 12CS0282 with Human Macrophages

Gentamicin protection assays were performed for quantitative analysis of invasion and survival of *C. ulcerans* 809, *C. silvaticum* W25, *C. pseudotuberculosis* 12CS0282 and *C. pseudotuberculosis* FRC41 in phagocytes. For this purpose, THP-1 cells, a human leukemia monocyte cell line, were differentiated by addition of 10 ng ml^−1^ phorbol 12-myristate 13-acetate. Cells were seeded in a density of 2 × 10^5^ cells per well with 500 μL medium without antibiotics 24 h prior to infection in 24-well plates. Bacteria used for infection of the cells were inoculated from overnight cultures to an optical density at 600 nm (OD_600_) of 0.2 in fresh medium and incubated at 37 °C and 125 rpm until an OD_600_ of 0.4–0.6 was reached. Cells were harvested by centrifugation (4500× *g*, 5 min, 4 °C) and washed with cold PBS under the same conditions. The cell pellet was resuspended in 500 μL cold PBS and the OD_600_ was determined in triplicates and adjusted to a value of 1 in 1000 μL PBS and finally diluted 10^−2^. 50 μL of this cell suspension in 450 μL prewarmed cell culture medium without antibiotics per well were used as inoculum which results in a multiplicity of infection (MOI) of 1. Serial dilutions of the inoculi were plated on blood agar plates (Oxoid, Wesel, Germany) using an Eddy Jet Version 1.22 (IUL Instruments, Barcelona, Spain) and incubated at 37 °C for two days. The infection plates were centrifuged for 5 min at 350× *g* to synchronize the infection and incubated at cell culture conditions for 30 min to allow phagocytosis of bacteria. Subsequently, the supernatant containing non-engulfed bacteria was aspirated, cells were washed once with PBS and remaining extracellular bacteria were killed by addition of 100 μg mL^−1^ gentamicin in cell culture medium. After 2 h, cells were either lysed by adding 500 μL of 0.1% Triton X-100 in PBS and intracellular bacteria were recovered by plating serial dilutions of the lysates on blood agar plates or further incubated for analysis at later time points (4 and 20 h). After incubation at 37 °C for two days, the number of colony forming units (CFU) was determined. The ratio of bacteria used for infection (number on colonies on the inoculum plates) and bacteria on the lysate (number of colonies on the lysate plates) multiplied with 100 gave the percentage of viable intracellular bacteria at different time points. When the survival of intracellular bacteria in THP-1 cells was analyzed over the time, the number of CFU at 2 h was set to 100% and later time points were calculated based on this value.

### 2.8. Statistical Considerations

For label-free quantification only proteins, which are present in all three independent biological replicates, were considered as identified. The peak areas of the identified proteins were normalized via the molecular weight and the relative abundance of each protein was calculated based on the total protein approach (TPA) method [29].

For gentamicin protection assays, experiments were performed in three independent biological replicates with technical triplicates (n = 9) and means and standard deviations were calculated. Unpaired Student’s *t*-test was performed using GraphPad Prism 7.0 (GraphPad, San Diego, CA, USA).

## 3. Results

### 3.1. Genome Analysis

#### 3.1.1. Phylogenomic Characteristics of Strain 12CS0282

The genome sequence of strain 12CS0282 is 2.3 Mb in size with a GC content of 52.2 mol%. 2119 coding sequences, 5 rRNA, 49 tRNA and 1 tmRNA were annotated.

**Figure 2:**
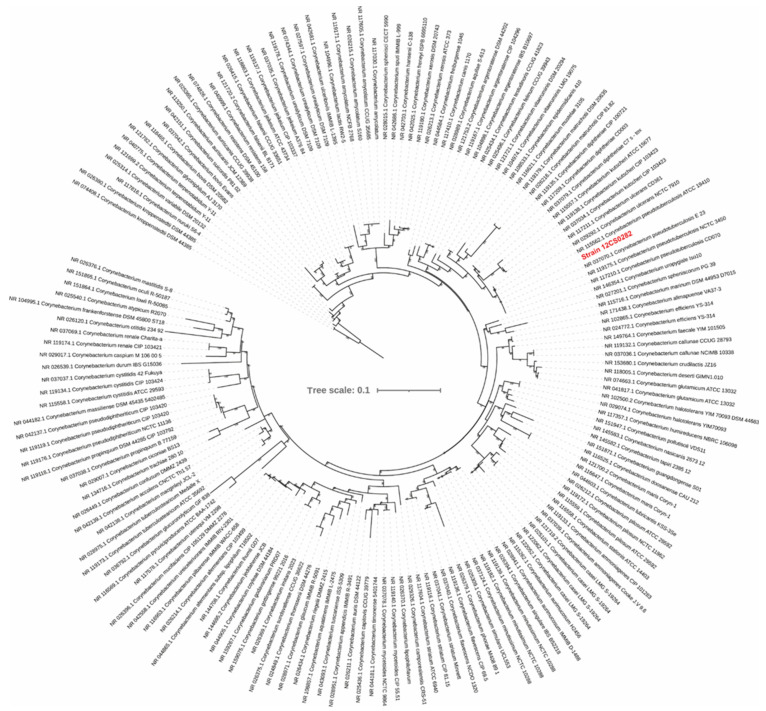
A maximum-likelihood tree from the 16S rRNA gene sequence alignment. The scale bar represents nucleotide substitution per site. Strain 12CS0282 is highlighted in red.

The phylogenetic tree calculated from the 16S rRNA sequences grouped strain 12CS0282 with other *C. pseudotuberculosis* strains (Figure 2). To further confirm the strain identity, we calculated digital DNA-DNA hybridization values between strain 12CS0282 and representatives of closely related corynebacterial species. The program GGDC 3.0 uses three approaches (formulas) for calculation of the dDDH values, but the values derived from formula #2 are recommended for the dataset that includes draft assemblies [23,53]. These values are summarized in Table 1. dDDH values between strain 12CS0282 and the type strain of *C*. *pseudotuberculosis* were above the 70% cut-off value to define species, confirming that strain 12CS0282 belongs to *C*. *pseudotuberculosis*. The dDDH values between this strain and other *Corynebacterium* species was <30% (Table 1).

**Figure 3:**
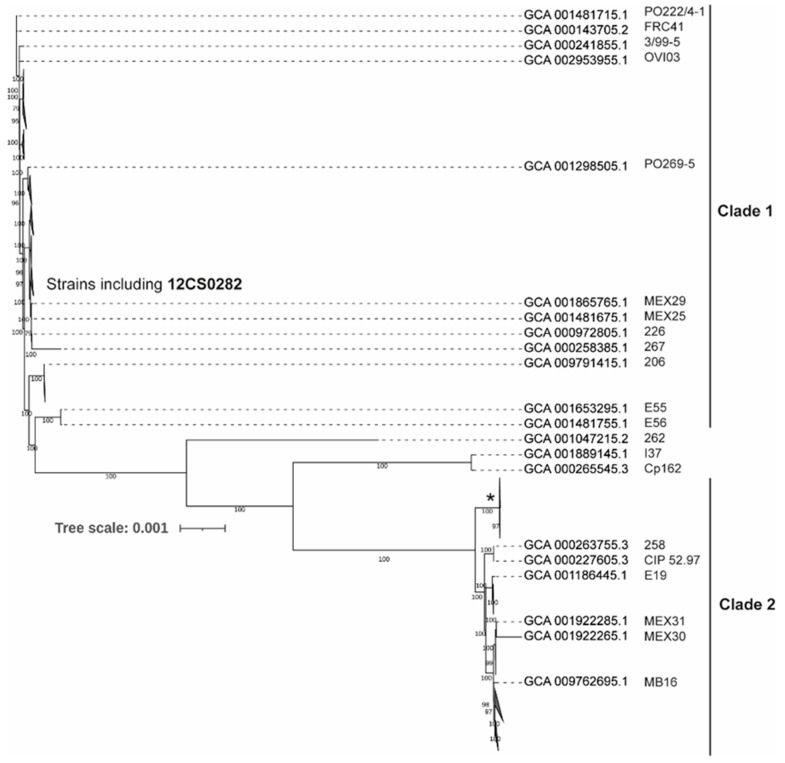
Maximum-likelihood tree from nucleotide sequence alignment of the core genome calculated from 131 *C. pseudotuberculosis* strains. Clades with the average branch distance to their leaves below 0.0001 were collapsed. Nine out of 11 strains in the clade marked by * possess the *tox* gene. The scale bar represents nucleotide substitution per site (see Supplementary Figure S1 for the all strain details).

A comparative analysis of 131 genomes indicated that approximately 2/3 of *C. pseudotuberculosis* genome is highly conserved genome with 1562 genes present in 99–100% strains. The size of the pan-genome is 3778 genes including 250 Soft core genes (95–99% strains), 620 Shell genes (15–95% strains) and 1346 Cloud genes (0- 15% strains). A phylogenetic tree from the core genome separated *C. pseudotuberculosis* strains into two major clades (Figure 3 and Figure S1). This is consistent with the previous studies [12,13]. Clade 1 include 95 strains including all strains identified as biovar *ovis* (Supplementary Figure S1). 33 strains grouped in Clade 2 and include most of the strains identified as biovar *equi* (Supplementary Figure S1). Three strains were quite distinct from both the clades, including one strain, Cp162, also identified as biovar *equi*. These observations are consistent with the findings of Soares and coworker [13].

#### 3.1.2. Virulence Genes in Strain 12CS0282

A protein BLAST search for the pilus gene clusters identified both *spaBC* (cp12CS0282_00823-cp12CS0282_00828) and *spaDEF* (cp12CS0282_00855- cp12CS0282_00860) type cluster in strain 12CS0282. However, cp12CS0282_00826 to cp12CS0282_00828 are partial SpaC sequences. Therefore, *spaC* is a pseudogene in this strain. The SpaDEF type cluster has all required genes in this strain [9]. Other virulence genes reported in *C. pseudotuberculosis* are also present in the strain 12CS0282 (Table 2), suggesting that this strain is well equipped to cause infection in sheep and other animals. The *tox* gene encoding diphtheria-like toxin is absent in this strain.

We also searched for the presence of these proteins among other 130 *C. pseudotuberculosis* strains and found that most of them are highly conserved across the dataset with minor exceptions (Supplementary Table S1). Corynebacterial protease CP40 (Cpp) is one of these exceptions as it is absent in 27 out of 130 strains (Supplementary Table S1). The majority of strains also lacked the toxin that was only present among nine strains in Clade 2.

Besides these virulence factors, a number of other proteins may be involved in the virulence of *C. pseudotuberculosis* and were detected in the data set. The genes encoding PknD (cp12CS0282_00131), a serine/threonine protein kinase involved in pathogenesis *of M. tuberculosis* [54], a hypothetical protein (cp12CS0282_00875) which is highly abundant in the *C. pseudotuberculosis* 12CS0282 and PknG (cp12CS0282_00897, serine/threonine protein kinase) a putative vaccine target [55] were also highly conserved within the data set.

### 3.2. Proteome Analyses

#### 3.2.1. *C. Pseudotuberculosis* 12CS0282 Whole Cell Proteome, Surface Fraction and Secreted Proteome Fraction

The theoretical proteome of *C. pseudotuberculosis* strain 12CS0282 comprises 2174 unique proteins of which 70% were located to the cytoplasm, 20% are membrane associated and 10% of the proteins are predicted to be secreted. Twenty-three percent include transmembrane helices (TMH), 5% a SPI secretion signal, 3% are secreted lipoproteins including a SPII signal sequence and 1% are secreted via a TAT secretion system.

**Figure 4:**
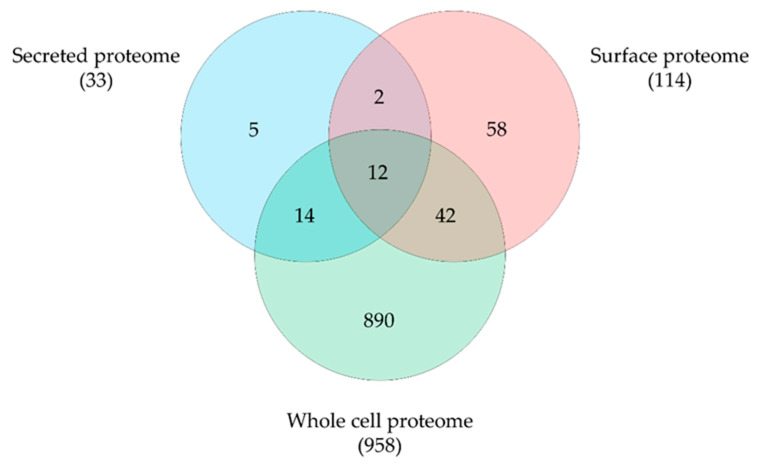
*C. pseudotuberculosis* 12CS0282 proteins identified in this study. Distribution and overlap of proteins in the different fractions analyzed. Data sets were curated using localization prediction approaches and cytoplasmic proteins were eliminated from surface and secreted proteome fractions.

By mass spectrometry, we were able to identify 1444 proteins, representing 66.4% of the theoretical proteome. A total of 1317 proteins were detected in the whole proteome, 1083 proteins in the surface fraction obtained by trypsin treatment and 187 proteins in the secreted proteins fraction with an overlap of 159 proteins. Obviously, the tryptic shaving procedure resulted in a high number of lysed cells, as described earlier for a *C. ulcerans* strain [31]. Therefore, data sets were curated using localization prediction approaches and cytoplasmic proteins were eliminated from surface and secreted proteome fractions. Furthermore, only proteins identified in all three runs were considered for further analysis. In total 1023 unique proteins were found, 958 proteins from the whole cell fraction, 114 proteins from the surface fraction and 33 secreted proteins were present in all three runs and 12 overlapping proteins were found (Figure 4).

#### 3.2.2. Metabolic Pathway Analysis

**Figure 5:**
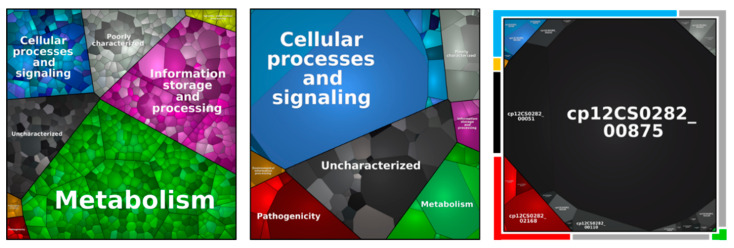
Metabolic pathways of identified proteins in the whole cell proteome (**left**), surface fraction (**middle**) and extracellular proteome (**right**). Each area displays one protein and equal to the relative abundance. Pathways are separated in metabolism (green), information storage and processing (purple), cellular processes and signaling (blue), environmental information processing (orange), genetic information processing (yellow), poorly characterized (grey), uncharacterized (black), and in pathogenicity (red).

The proteins identified were classified in respect to their metabolic function (Figure 5, Table 3). When the proteins of the secretome were analyzed in detail, protein Cp12CS0282_00875 represented 69.9 ± 3.1% of the total protein content. This putative trypsin-like serine protease has a homology with Vsp2, a known virulence factor from *C. ulcerans*, and is highly conserved within the analyzed *C. pseudotuberculosis* strains (Supplementary Table S1).

Independent from the pathway, a similar percentage of the theoretical proteome and the identified proteins was found, while proteins related to pathogenicity and poorly and uncharacterized proteins were slightly over-represented. The theoretical proteome and the overall identified protein pool included 3.1% of protein related to pathogenicity, while 3.9% of proteins found in all biological replicates were attributed to this group. Moreover, a high number of poorly characterized and uncharacterized proteins were found, summing up to 42.6% of the theoretical proteome, 45.7% of all identified proteins and 46.0% of proteins found in all replicates.

In respect to the main metabolic pathways, all proteins from the glycolysis, pentose-phosphate pathway, synthesis of the cell envelope, glycolysis, gluconeogenesis, tricarboxylic acid (TCA) cycle, heme biosynthesis, fatty acid synthesis and amino acid synthesis were found except enzymes involved in the synthesis of phenylalanine and tyrosine.

#### 3.2.3. Identification of Virulence Proteins

The respective proteins of almost all annotated virulence genes identified in the 12CS0282 genome were identified in different proteome fractions when the extracellular secretome fraction, cell-wall-bound surface fraction and whole cell fraction were analyzed (Table 4), indicating that this strain is already preadapted to host contact and pathogenicity-related proteins do not have to be expressed upon host contact.

#### 3.2.4. Reverse Vaccinology

From the cellular and extracellular proteome of *C. pseudotuberculosis* strain 12CS0282 only experimentally identified proteins from the surface fraction and secreted proteome were considered for further analyses in a reverse vaccinology approach. The theoretical proteome of *C. pseudotuberculosis* strain 12CS0282 was filtered for proteins located on the surface and secreted proteins. From these, only proteins from the core proteome of *C. pseudotuberculosis* were selected for prediction of probable vaccine targets (Vaxign2), antigenic potential (VaxiJen) and filtered for non-host homologous proteins and essential proteins. The described reverse vaccinology approach (Figure 1) revealed 22 probable vaccine and drug targets (Table 5). Four of these, Ndh (cp12CS0282_01097), SenX3 (cp12CS0282_00370), FtsI (cp12CS0282_01491) and YidC (cp12CS0282_00093) were also identified as putative vaccines targets in a former in silico analysis of *C. pseudotuberculosis* [45].

**Figure 6:**
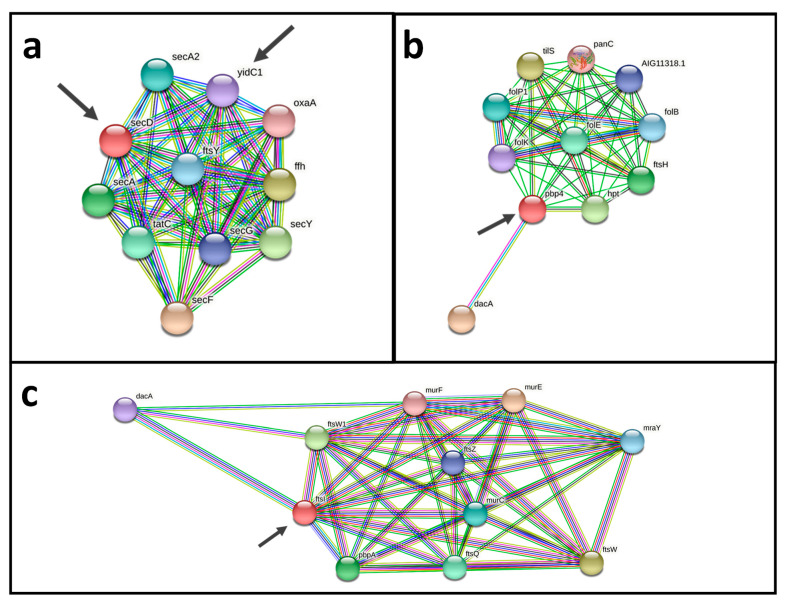
Protein-protein interaction networks of putative vaccine and drug targets. (**a**) The two probable vaccine and drug targets SecD (cp12CS0282_01259) and YidC1 (cp12CS0282_00093), have direct interactions and are interacting with components of bacterial protein secretion (Sec proteins) and cell division (Fts proteins). (**b**,**c**) Pdp4 (cp12CS0282_00932) and FtsI (cp12CS0282_01491) show interactions with DacA, Fts proteins and Mur proteins, which are involved in cell wall synthesis and cell division. Putative vaccine and drug targets identified here are indicated by arrows.

An analysis using a protein-protein interactions network revealed promising vaccine and drug targets (Figure 6). Both potential vaccine and drug targets Pdp4 (cp12CS0282_00932) and FtsI (cp12CS0282_01491) show interactions with DacA, Fts proteins and Mur proteins. Fts and Mur proteins are involved in construction of the cell wall and for cell division (Figure 6a,b). Two probable vaccine and drug targets have a direct interaction SecD (cp12CS0282_01259) and YidC1 (cp12CS0282_00093). Both proteins are involved in bacterial secretion (Sec proteins) and cell division (Fts proteins) (Figure 6c).

#### 3.2.5. Interaction with Macrophages

**Figure 7:**
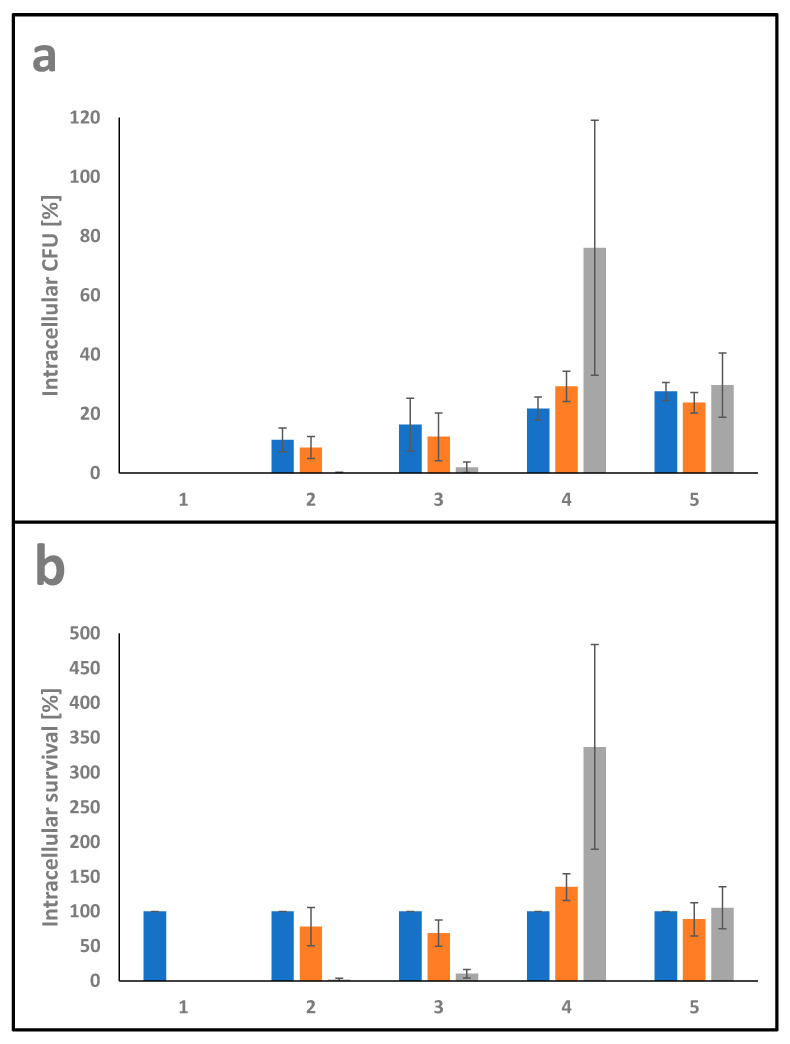
Quantitative analysis of *C. pseudotuberculosis*–macrophage interaction. THP-1 cells were infected with *C. ulcerans* 809 (2), *C. silvaticum* W25 (3), *C. pseudotuberculosis* 12CS0282 (4) and *C. pseudotuberculosis* FRC41 (5) at MOI 1 for 30 min. Infection with the non-pathogenic *C. glutamicum* ATCC13032 (1) served as a negative control. In order to kill extracellular bacteria, cells were incubated with medium containing gentamicin and after 2 (blue), 4 (orange) and 20 h (grey), cells were harvested, lysed and lysates were plated on blood agar plates to recover intracellular CFU. (**a**) Intracellular CFU in percent referred to the inoculum. (**b**) Intracellular survival in percent referred to the bacteria that were taken up after 2 h.

The ability of pathogens to invade and survive in host cells is fundamental for host colonization. In order to determine intracellular viability in THP-1 cells, gentamicin protection assays were carried out using *C. glutamicum* ATCC13032 as a non-pathogenic corynebacterium, *C. ulcerans* 809 and *C. silvaticum* W25 as pathogenic members of the genus together with *C. pseudotuberculosis* strains 12CS0282 and FRC41. THP-1 cells were infected with bacteria at MOI 1 for 30 min. Subsequently, the cells were washed and further incubated for 2, 4 and 20 h in cell culture medium containing gentamicin to kill extracellular bacteria, then detached and lysed. The ratio of colony forming units (CFU) in inoculum and lysates provided the percentage of invasive bacteria (Figure 7a). Furthermore, the ratio of internalized viable bacteria (after 2 h infection) and CFU from lysates from 4 h and 20 h infections gave the percentage of time-dependent survival within the phagocytes (Figure 7b).

The intracellular survival rate of the non-pathogenic *C. glutamicum* ATCC13032 was 0% after 2 h incubation, indicating that *C. glutamicum* was not able to resist phagolysis by THP-1 cells and is quickly eradicated. *C. ulcerans* 809 had the lowest uptake rate with 11 ± 4% followed by *C. silvaticum* W25 with 16 ± 9% on average. The *C. pseudotuberculosis* strains reached values between 22 ± 4% and 28 ± 3%.

The CFU of *C. ulcerans* and *C. silvaticum* gradually decreased with a survival rate of about 78 ± 28% and 69 ± 19% after 4 h incubation and only 2 ± 2% and 10 ± 6% after 20 h incubation, respectively. While *C. pseudotuberculosis* strain FRC41, isolated from a 12 years old girl with necrotizing lymphadenitis, did not multiply in the macrophages, strain 12CS0282 showed significant growth inside the human phagocyte cells used with survival rates of 135 ± 19% after 4 h incubation and 337 ± 147% on average after 20 h of incubation.

## 4. Discussion

Although *C. pseudotuberculosis* is a member of the toxigenic corynebacteria [2], sequences of *tox* gene-encoding corynephages are rarely detectable in the available genomes. Less than 10% of the strains are *tox* gene carriers. Nevertheless, the species is an important animal pathogen and obviously also a potential danger for human health.

The *C. pseudotuberculosis* strain 12CS0282 investigated here showed a higher resistance against human phagocytes than pathogenic *C. ulcerans* [56,57], *C. pseudotuberculosis* FRC41 [9] and *C. silvaticum* [58] strains. Strain 12CS0282 was not only able to survive for more than 20 h within human macrophages, but could even multiply within the phagocytes. Either the strain is able to survive the harsh conditions within the macrophage, or it is able to inhibit phagolysosome maturation as shown for *C. diphtheriae* and *C. ulcerans* [59,60]. A prime candidate for macrophage damage is the sphingomyelinase phospholipase D (PLD), which was shown to promote survival of *C. pseudotuberculosis* in a murine macrophage cell line [61]. Another possible candidate is the trypsin-like serine protease Cp12CS0282_00875, which is the by far most abundant secreted protein. However, although intriguing, it is most likely not a single virulence factor, but a combination of proteins and liposaccharides may determine the pathogenic potential of the bacteria, since virulence-related processes such as adhesion, invasion and intracellular survival seem to be multifactorial mechanisms in corynebacteria [62].

As demonstrated by proteome analyses, almost all known virulence factors for *C. pseudotuberculosis* were expressed already under standard laboratory conditions and without any host contact, indicating a high degree of pre-adaptation to host conditions putatively also enabling fast response to phagocytosis and supporting survival within the macrophages. As an approach to get further information on proteins important for host cell interaction and survival in macrophages proteome analyses of bacteria internalized by phagocytes may be carried out in future studies.

In the light of the pathogenic potential of the species, vaccination may be the gold standard to inhibit infection and colonization of host species and different targets were already published [13,14,15]. To contribute to this field, we used a reverse vaccinology approach similar to a study of Araújo and co-workers [45] and were able to validate four the previously identified targets [45] also for strain 12CS0282. The reverse vaccinology approach carried out here hint to especially three promising putative vaccine targets, FtsI, SecD and YidC. FtsI is a transpeptidase required for synthesis of peptidoglycan, involved in cell cycle control and cell division of growing bacteria and predicted to be a potential drug target in *M. tuberculosis* [63]. SecD is involved in protein secretion in *M. tuberculosis* [64] and contributes to the virulence potential in *Listeria monocytogenes* [65] and *Staphylococcus aureus* [66]. YidC is an essential insertase protein, which mediates the assembly and insertion of inner membrane proteins. The function is related to folding and insertion of lipoproteins in the plasma membrane [67] and controls respiratory metabolism in *M. tuberculosis* [68]. In addition, the identified proteins may serve as excellent drug targets, since they interfere with cell envelope synthesis and protein secretion. As our dataset was filtered for essential proteins, it is a shortcoming of this study that these targets cannot be easily verified by gene deletion experiments. Either methods for gene knock-downs have to be developed for this organism, or proteins have to be overexpressed, purified and used for antibody production to test their suitability in animal studies, as descried earlier for *C. pseudotuberculosis* proteins Cp09 and Cp40 [14].

As an alternative to the described proteome and reverse vaccinology approach, immunopeptidomics may help to develop vaccines directed against *C. pseudotuberculosis*. Immunopeptidomics approaches focus on antigen discovery by the detection of peptides that are presented at the surface of cells by major histocompatibility complexes. In principle, this allows an untargeted identification of bacterial antigens. However, up to now, only a few-mainly intracellular-bacterial pathogens have been investigated by this approach. The reason for this is the main drawback of the technique, the large number of infected cells required for proteome analysis [69].

In summary, our study provides a further example of the strength of combining different -omics and bioinformatics approaches as demonstrated earlier [45] and may help to develop strategies to combat this important animal pathogen, which also has a pathogenic potential against humans.

## Figures and Tables

**Table 1:** Digital DNA-DNA hybridization values between strain 12CS0282 and representative strains of closely related species.

Reference Genome	DDH	Distance	G + C Difference
*C. belfanti* DSM 105776 ^T^	20.6	0.2129	1.44
*C. diphtheriae* DSM44123 ^T^	20.8	0.2116	1.35
*C. pseudotuberculosis* ATCC 19410 ^T^	99.9	0.0003	0
*C. pseudotuberculosis* DSM 20689 ^T^	99.9	0.0002	0
*C. silvaticum* KL0182 ^T^	28.5	0.1509	2.26
*C. silvaticum* W25	28.5	0.1509	2.25
*C. ulcerans* FRC11	27.6	0.1563	1.17
*C. ulcerans* NCTC 7910 ^T^	27.5	0.1568	1.13

**Table 2:** Presence of known virulence genes in strain 12CS0282.

Virulence Gene	Function	Identifier in Strain 12CS0282
cpfrc_00029 (*pld*)	phospholipase D (sphingomyelin-degrading enzyme)	cp12CS0282_00124
cpfrc_00128 (*nor*)	nitric oxide reductase	cp12CS0282_00230
cpfrc_00386 (*nanH*)	neuraminidase H (sialidase)	cp12CS0282_00502
cpfrc_00397	secreted subtilisin-like serine protease	cp12CS0282_00513
cpfrc_00491 (*dtsR2*)	acyl-CoA carboxylase b-subunit involved in mycolic acid synthesis	cp12CS0282_00606
cpfrc_00492 (*dtsR1*)	acetyl-CoA carboxylase b-subunit involved in fatty acid synthesis	cp12CS0282_00607
cpfrc_00536	secreted SGNH-hydrolase	cp12CS0282_00649
cpfrc_00562	secreted trypsin-like serine protease	cp12CS0282_00678
cpfrc_00565 (*nrpS1*)	nonribosomal peptide synthetase 1	cp12CS0282_00682
cpfrc_00594 (*rpfA*)	resuscitation-promoting factor A (muralytic enzyme)	cp12CS0282_02168
cpfrc_00679 (*rpfB*)	resuscitation-promoting factor B (muralytic enzyme)	cp12CS0282_02083
cpfrc_01079 (*rpfI*)	resuscitation-promoting factor interacting protein (D,L-endopeptidase)	cp12CS0282_01163
cpfrc_01634	secreted subtilisin-like serine protease	cp12CS0282_01728
cpfrc_01801	nonribosomal peptide synthetase 2	cp12CS0282_00926
cpfrc_01895 (*cpp*)	corynebacterial protease CP40 (serine protease)	cp12CS0282_00833
cpfrc_01953 (*accD3*)	acyl-CoA carboxylase b-subunit involved in mycolic acid synthesis	cp12CS0282_00773

**Table 3:** Metabolic pathway analysis.

Pathway	Theoretical Proteome	Identified Proteins	n = 3
Cellular processes and signaling	235 [11.1%]	141 [9.8%]	97 [9.5%]
Environmental information processing	67 [3.2%]	37 [2.6%]	25 [2.4%]
Genetic information processing	52 [2.5%]	38 [2.6%]	24 [2.3%]
Information storage and processing	224 [10.6%]	142 [9.8%]	100 [9.8%]
Metabolism	563 [26.6%]	382 [26.5%]	267 [26.1%]
Pathogenicity	53 [3.1%]	45 [3.1%]	40 [3.9%]
Poorly characterized	253 [11.9%]	187 [13.0%]	142 [13.9%]
Uncharacterized	671 [31.7%]	472 [32.7%]	328 [32.1%]
Total	2118	1444	1023

**Table 4:** Validation and distribution of *C. pseudotuberculosis* 12CS0282 virulence factors. Proteins encoded by virulence genes in Table 1 were analyzed in respect to presence, localization (E, extracellular, S, surface; W, whole cell fraction) and relative abundance (% of protein content in fraction).

Designation	Function	Localization and Relative Abundance
cp12CS0282_00124 (*pld*)	phospholipase D	E (0.5%), W (0.4%)
cp12CS0282_00230 (*nor*)	nitric oxide reductase	-
cp12CS0282_00502 (*nanH*)	neuraminidase H	S (0.6%)
cp12CS0282_00513	secreted subtilisin-like serine protease	-
cp12CS0282_00606 (*dtsR2*)	acyl-CoA carboxylase b-subunit involved in mycolic acid synthesis	W (0.5%)
cp12CS0282_00607 (*dtsR1*)	acetyl-CoA carboxylase b-subunit involved in fatty acid synthesis	W (0.4%)
cp12CS0282_00649	secreted SGNH-hydrolase	S (6.3%), W (0.2%)
cp12CS0282_00678	secreted trypsin-like serine protease	E, S, W
cp12CS0282_00682 (*nrpS1*)	nonribosomal peptide synthetase 1	-
cp12CS0282_02168 (*rpfA*)	resuscitation-promoting factor A (muralytic enzyme)	E (4.2%)
cp12CS0282_02083 (*rpfB*)	resuscitation-promoting factor B (muralytic enzyme)	S (0.3%)
cp12CS0282_01163 (*rpfI*)	resuscitation-promoting factor interacting protein (D,L-endopeptidase)	-
cp12CS0282_01728	secreted subtilisin-like serine protease	E (0.04%), S (0.3%), W (0.2%)
cp12CS0282_0092	nonribosomal peptide synthetase 2	-
cp12CS0282_00833 (*cpp*)	corynebacterial protease CP40	S (0.2%)
cp12CS0282_00773 (*accD3*)	acyl-CoA carboxylase b-subunit involved in mycolic acid synthesis	W (0.6%)

**Table 5:** Predicted vaccine and drug targets. The table shows the protein ID of the corresponding protein from *C. pseudotuberculosis* strain 12CS0282, the protein name, molecular weight (MW) and predicted stability (+: stable; −: unstable).

Protein ID	Protein Name	MW (Da)	Stability
cp12CS0282_00093	membrane protein insertase	36,402.13	−
cp12CS0282_00370	signal-transduction histidine kinase	44,858.97	+
cp12CS0282_00394	cytochrome C biogenesis protein	60,045.54	+
cp12CS0282_00666	hypothetical protein	109,088.9	+
cp12CS0282_00740	putative cell wall biosynthesis protein	46,819.89	−
cp12CS0282_00766	diacylglycerol acyltransferase/mycolyltransferase	36,582.47	+
cp12CS0282_00770	hypothetical protein	32,832.33	+
cp12CS0282_00932	hypothetical protein	41,309.86	+
cp12CS0282_00991	adaptive-response sensory-kinase	54,403.91	−
cp12CS0282_01097	NADH dehydrogenase-like protein	49,062.65	+
cp12CS0282_01233	endolytic murein transglycosylase	41,096.36	+
cp12CS0282_01244	FMN reductase	19,296.33	−
cp12CS0282_01259	protein translocase subunit	65,933.18	+
cp12CS0282_01491	penicillin-binding protein	73,053.21	+
cp12CS0282_01513	cytochrome Bc1 complex cytochrome B subunit	110,964	+
cp12CS0282_01515	cytochrome Bc1 complex cytochrome C subunit	31,394.71	+
cp12CS0282_01518	cytochrome C oxidase subunit 2	40,192.72	+
cp12CS0282_01584	bifunctional protein	41,939.33	+
cp12CS0282_01590	hypothetical protein	34,917.49	−
cp12CS0282_01839	hypothetical protein	34,482.87	−
cp12CS0282_02102	signal transduction histidine-protein kinase/phosphatase	56,585.5	−
cp12CS0282_02120	hypothetical protein	24,721.76	+

## Data Availability

The genome sequences of strains *C. pseudotuberculosis* 12CS0282 has been submitted to GenBank (Accession no.: NZ_CP074370.1). Raw data files and msf files of mass spectrometric analyses were deposited to the ProteomeXchange Consortium (http://proteomecentral.proteomexchange.org) via the PRIDE partner repository [70]. Data are available via ProteomeXchange PXD036354 (project webpage: http://www.ebi.ac.uk/pride/archive/projects/PXD036354; FTP download: ftp://ftp.pride.ebi.ac.uk/pride/data/archive/2022/11/PXD036354; published 21 November 2022).

## Supplementary Materials

The following supporting information can be downloaded at: https://www.mdpi.com/article/10.3390/proteomes10040039/s1, Figure S1: Phylogenetic tree of *C. pseudotuberculosis*. Table S1: Distribution of virulence genes in *C. pseudotuberculosis*. Table S2: Mass spectrometry data of 12CS0208 proteins.
