# Supplementary figures and images for: Multi-Omics of Corynebacterium Pseudotuberculosis 12CS0282 and an In Silico Reverse Vaccinology Approach Reveal Novel Vaccine and Drug Targets
Source: Proteomes. 2022 Nov 23;10(4):39. doi: 10.3390/proteomes10040039 (PMC9784263; doi:10.3390/proteomes10040039)

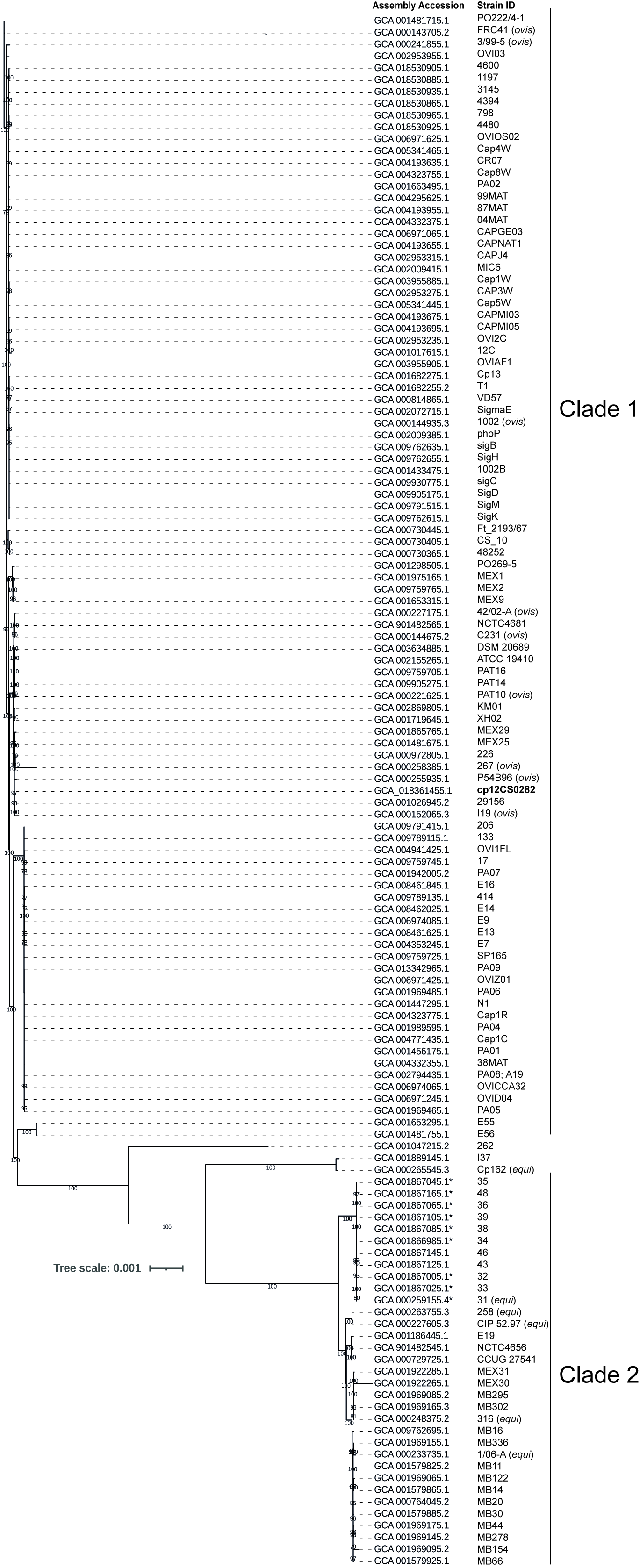

Supplement: Supplementary file 1 [file proteomes-10-00039-s001.zip › Supplementray Figure S1.tif]
